# ShK-Domain-Containing Protein from a Parasitic Nematode Modulates *Drosophila melanogaster* Immunity

**DOI:** 10.3390/pathogens11101094

**Published:** 2022-09-24

**Authors:** Aklima K. Lima, Harpal Dhillon, Adler R. Dillman

**Affiliations:** Department of Nematology, University of California, Riverside, CA 92507, USA

**Keywords:** parasitic nematode, ESPs, toxin, immunomodulator, ShK, fruit fly, negative geotaxis, chill coma recovery

## Abstract

A key component to understanding host–parasite interactions is the molecular crosstalk between host and parasite. Excreted/secreted products (ESPs) released by parasitic nematodes play an important role in parasitism. They can directly damage host tissue and modulate host defense. Steinernema carpocapsae, a well-studied parasite of insects releases approximately 500 venom proteins as part of the infection process. Though the identity of these proteins is known, few have been studied in detail. One protein family present in the ESPs released by these nematodes is the ShK family. We studied the most abundant ShK-domain-containing protein in *S. carpocapsae* ESPs, Sc-ShK-1, to investigate its effects in a fruit fly model. We found that Sc-ShK-1 is toxic under high stress conditions and negatively affects the health of fruit flies. We have shown that Sc-ShK-1 contributes to host immunomodulation in bacterial co-infections resulting in increased mortality and microbial growth. This study provides an insight on ShK-domain-containing proteins from nematodes and suggests these proteins may play an important role in host–parasite interactions.

## 1. Introduction

Parasitic nematodes continue to cause significant morbidity and mortality to humans and animals. Often these parasites co-occur with other pathogens, worsening the progression of these diseases [1]. The resulting suffering ranges from innocuous to severe. While parasitic nematodes are usually viewed in a negative light, recent studies suggest that worms play an important role in promoting human health and proper immune development [2,3]. Probiotic worm therapy has a promising effect against inflammatory bowel diseases [2,4,5]. Helminths are also proving to be a rich source of interesting and useful molecules that could lead to novel treatments in the field of medicine [1,6]. Worm-dependent treatments are thought to be important in suppressing cytokines and regulating T cells that contribute to the therapy of many autoimmune diseases, e.g., multiple sclerosis, ulcerative colitis, etc. [2,7,8]. There are also well-known beneficial parasitic nematodes that are used for biologic control of agricultural pests. Entomopathogenic nematodes (EPNs) are used in integrated pest management [9,10,11]. EPNs are also regarded as a model system and are widely used in evolutionary biology, biological control, symbiosis, soil ecology, and neurobiology [12,13,14,15,16].

Similar to other parasitic nematodes, EPNs release excreted/secreted products (ESPs) into the host tissue soon after entering the host’s body [14,17,18]. ESPs are a complex mixture of proteins, small molecules and nucleic acids that facilitate parasite survival in the host environment and weaken the host defense system [14]. ShK-domain-containing proteins are present in the ESPs of many parasitic nematodes [19]. ShK-domain-containing proteins are named after a protein in the venom of the sea anemone *Stichodactyla helianthus* where they were first identified [4,7,19]. Later ShK-domain-containing proteins were found in various animal venoms [20]. The proteins from the ShK superfamily are known for different biologic functions including neurotoxic, immunosuppressive, paralytic, and hemolytic functions [20,21,22]. Many are known to inhibit voltage-gated and calcium-activated K^+^ channels [2,4,23]. Studies show that ShK peptides from parasitic nematodes block Kv1.3 channels in human T cells and suppress IFNγ production [2,4,7]. ShK peptides also suppress the proliferation of rat CCR7 effector memory T cells and inhibit delayed type hypersensitivity, which is a cell-mediated immune response [2,24]. Due to its effect on effector T cells, this protein became an attractive therapeutic candidate in treating autoimmune diseases [2,21]. One of the ShK-domain-containing proteins from sea anemone was developed as the drug Dalazatide and is currently under phase 1b clinical trials for the treatment of plaque psoriasis, a common autoimmune skin lesion [4,25,26,27,28].

ShK-domain-containing proteins are present in animals and plants, yet few studies have been done with these proteins in nematodes. Understanding the role of the nematode ShK proteins may increase our understanding of how nematodes modulate host immunity during infection. In this study, we characterized the functional role of an ShK protein from an insect parasitic nematode, *Steinernema carpocapsae*. Among the 14 ShK proteins secreted by *S. carpocapsae* during active infection [14], we chose to study the most abundant ShK protein, Sc-ShK-1 (Wormbase Parasite Gene ID-L596_029213) found in the ESPs. The Sc-ShK-1 has four ShK domains and the size of whole protein is about 20 KD. To characterize the protein, a recombinant version of Sc-ShK-1 was generated using a yeast expression system and purified by chromatographic techniques. Using the fruit fly, we tested in vivo activity of the protein and assessed fly health by measuring survival and behavior. The fruit fly is a powerful model organism and studying the function of Sc-ShK-1 in this system allowed us to use large numbers of animals [29]. We injected Sc-ShK-1 into fruit flies and observed toxicity effects under several conditions. To evaluate the fly health in non-lethal way, we assessed chill coma recovery and negative geotaxis. Further, we tested the immunomodulatory role of the protein using *Streptococcus pneumoniae* infections and found that Sc-ShK-1 decreases fly resistance to infection leading to decreased survival and increased bacterial growth. Collectively, these data suggest that Sc-ShK-1 from *S. carpocapsae* plays an important role in modulating host immunity and/or decreasing host health during nematode infection. 

## 2. Materials and Methods

### 2.1. Generation of Recombinant Protein

The full length *Sc-Shk-1* gene sequence was retrieved from the Wormbase Parasite (L596_029213). The synthesis of gene and recombinant expression in *Pichia pastoris* was performed by Bio-Basic Inc (https://www.biobasic.com/, accessed on 16 July 2021). The gene was optimized for yeast expression and synthesized artificially. The optimized gene was cloned in pPIC9K *Pichia pastoris* (yeast) expression vector in EcoRI and NotI restriction sites with a proprietary tag from Bio-Basic Inc and an N-terminal histidine tag followed by SUMO-STAR protein. Sc-ShK-1 protein was expressed recombinantly in *Pichia pastoris*. The recombinant protein was first purified by affinity purification (Ni-NTA) followed by size exclusion chromatography using Superdex 75 size exclusion column (Figure S1A,B). The fusion protein was digested with sumo star protease and Sc-Shk-1 protein was purified by size exclusion chromatography (Figure S1C). The Western blot analysis was performed to confirm the expression of Sc-Shk-1 (Figure S1D).

### 2.2. Fly Breeding and Maintenance

OregonR strain of flies were grown on D2 glucose medium from Archon Scientific (Durham, NC, USA) and kept at 25 °C with 50% humidity on a 12 h light and 12 h dark cycle. 20 male and female adult flies were placed on a media bottle to start breeding. After 3 days the flies were removed from the media to maintain their age range. After 12 days, male flies were sorted in vials in group of 33 flies. Adult male flies aged 5–7 days were used for all the experiments.

### 2.3. Fly Injections for Toxicity Assays

Flies were anesthetized with CO_2_ and were injected with specific doses of Sc-ShK-1, MgTx, Denatured Sc-ShK-1, or buffer only in a total volume of 50 nL using a MINJ-FLY high-speed pneumatic injector (Tritech Research, Los Angeles, CA, USA) and individually pulled calibrated glass needles. Flies were injected into the abdomen close to where the thorax meets and slightly ventral from the dorsal-ventral cuticle axis, easily visible below the haltere. For starvation and desiccation conditions, injected flies were placed in empty vials with no food and water for 12 h. The number of dead flies was recorded hourly for 12 h. After 12 h, the flies were transferred to vials with food and survival was recorded daily. For starvation conditions, injected flies were placed in empty vials with water-soaked filter paper for 12 h. The number of dead flies were recorded hourly for 12 h. After 12 h flies were transferred to vials with food and survivals were recorded daily. For normal conditions, injected flies were directly placed in vials with fly food and survival was recorded daily. Each experiment was done with total 180 flies triplicated. Statistical analyses were done with GraphPad Prism software using Kaplan–Meier survival curves as log-rank analysis (Mantel-Cox). Log-rank test *p*-value significance indicated by asterisks on following cut offs values: 0.0004–0.0006 *** and <0.0001 ****.

### 2.4. Negative Geotaxis Assay

These assays were performed as previously described [29]. Briefly, flies were injected with specific doses of Sc-ShK-1, *S. pneumoniae*, MgTx, denatured Sc-ShK-1, or buffer only and placed in vials with food for overnight. The following day, flies were transferred to glass vials in groups of 15 flies each at room temperature. The glass vials were placed in a wooden frame to keep them upright. After a fifteen to twenty minutes of acclimation, the assay was performed. The glass vials were tapped three times and climbing was recorded using a video camera. The distance flies climbed in 5 s was determined from the video recording. Experiment was done with total 180 flies for each condition. Statistical analyses were done in Prism (GraphPad, San Diego, CA, USA) using Mann–Whitney test and an overall *p* value was calculated (** *p* < 0.05).

### 2.5. Chill Coma Recovery

These assays were performed as previously described [29,30]. Flies were injected with specific doses of Sc-ShK-1, *S. pneumoniae*, MgTx, denatured Sc-ShK-1, or buffer only and placed in vials with food overnight. The following day, flies were transferred to empty vials which were placed in bucket of ice for three hours to induce a chill coma. After 3 h, flies were transferred to a 24-well plate with one fly in each well. A timer was started when the plate with flies was removed from the ice. Time was marked when each individual fly was able to stand, which was considered recovery. Each fly was checked once per minute until recovered. Experiment was done with total 180 flies for each condition. Statistical analyses were done in Prism (GraphPad) and recovery curves of each condition were compared to PBS control flies using a Logrank test (Mantel-Cocx test). *p*-value (<0.0001 ****) significance indicated by asterisks.

### 2.6. Bacterial Co-Injection, Survival, and CFUs

*Streptococcus pneumoniae* bacterial infections were performed as previously described [31]. The dose of bacteria was titrated such that 20% of flies died in the first 2–7 days post infection, which is the LD_20_ dose. A dose of approximate 3000 CFUs (LD_20_) were injected into adult male fruit flies, which were 5–7 days old. The flies were placed them in vials with food in groups of 34 and kept at 29 °C. The same dose of bacteria along with 100 ng of either Sc-ShK-1 or denatured Sc-ShK-1 protein were co-injected into fruit flies, which were then placed in vials with food in a group of 34 flies at 29 °C. Survival recordings were taken daily. Each experiment was done with total 180 flies triplicated. Statistical analyses were done with GraphPad Prism software using Kaplan–Meier survival curves as log-rank analysis (Mantel-Cox). Log-rank test *p*-value (0.0018 **) significance indicated by asterisks. To calculate the initial and 24-h CFUs, 8 flies were taken from each condition just after injection and Day 1 post-injection. Flies were homogenized in 200 μL of PBS, serially diluted and plated on the appropriate agar plates and incubated overnight. Colonies were counted the next day. A total of 24 flies were used for each condition. Statistical analyses were performed by GraphPad prism shown unpaired *t*-test with error bar indicating mean with SEM and *p* value calculated 0.0379 *.

## 3. Results

### 3.1. S. carpocapsae Releases Sc-ShK-1 Protein in Hosts during Infection

**Figure 1:**
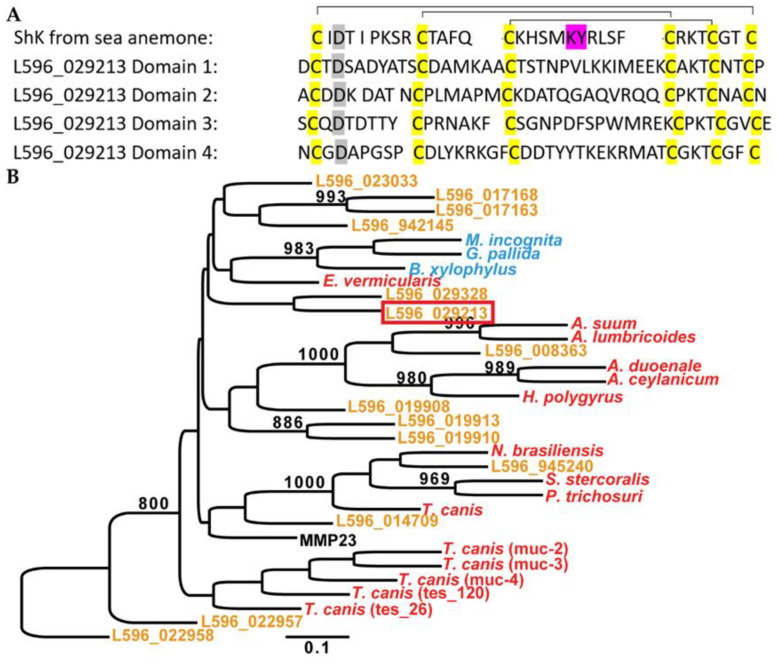
(**A**) Sequence alignment of an ShK peptide from sea anemone (uniprot accession ID-P29187) and four domains of Sc-ShK-1. ShK domains are residue of 35-37 aa with six conserved cysteines (highlighted in yellow) that putatively form three disulfide bonds between C1-C6, C2-C4 and C3-C5 (highlighted in black line). The K^+^ channel blocking residue Lys-Tyr (KY, highlighted in purple) is present in the sea anemone ShK domain, absent in Sc-ShK1 four domains. (**B**) A gene tree of 14 ShK proteins from *S. carpocapsae* ESPs and ShK proteins from other parasitic nematodes (modified from [14]. Sc-ShK-1 is highlight in a red box.

*Steinernema carpocapsae* releases 14 ShK-domain-containing proteins during active infection [14]. ShK-domain-containing proteins have one or more ShK domains, and each domain is 35-37 amino acid residues in length and contains six cysteines [21,32,33]. The cysteines are highly conserved among the domains and form three disulfide bonds between C1-C6, C2-C4, and C3-C5 connectivity (Figure 1A) [4]. ShK-domain-containing proteins can inhibit K^+^ channel function, which was thought to be regulated by a functional Lys-Tyr dyad between the third and fourth cysteines [2,4,7]. However, it has been shown that ShK-domain-containing proteins that lack these residues can still inhibit K^+^ channel function [34].

A gene tree was constructed with the 14 ShK proteins from *S. carpocapsae* ESPs and ShK-domain-containing proteins from other parasitic nematodes (modified from [14]). The proteins from *S. carpocapsae* have high sequence similarity with proteins from nematode parasites of vertebrates, such as *Ancylostoma*, *Ascaris*, *Toxocara*, and *Strongyloides* (Figure 1B). This suggests that ShK proteins are conserved among nematodes. Understanding the function of ShK-domain-containing proteins from *S. carpocapsae* will provide insight into the function of ShK proteins from nematode parasites of vertebrates including humans. Among the 14 ShK proteins in the *S. carpocapsae* ESPs, we selected the most abundant, which we named (Sc-ShK-1 (L596_029213), for further study. Sc-ShK-1 has four ShK domains: Sc-ShK-1 domain 1, Sc-ShK-1 domain 2, Sc-ShK-1 domain 3, and Sc-ShK-1 domain 4. Figure 1A shows a sequence alignment of an ShK-domain-containing protein from sea anemone (uniport accession ID-P29187) and Sc-ShK-1. The Sc-ShK-1 protein is missing the canonical K+ channel blocking residues in all the domains, however, the necessity of these residues in blocking K+ channel activity remains unclear [34].

### 3.2. Sc-ShK-1 Is Toxic under Starvation and Desiccation Conditions

**Figure 2:**
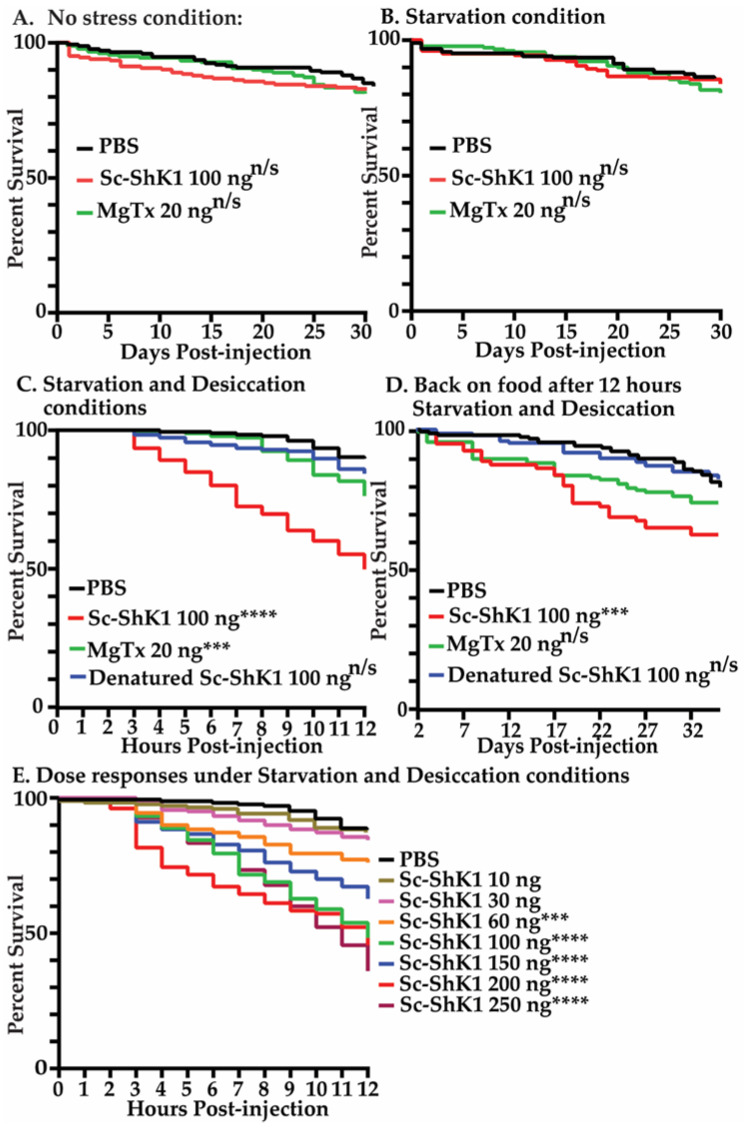
100 ng of recombinant Sc-ShK-1 was injected into fruit flies. (**A**) Survival was recorded under no stress conditions. (**B**) Survival was recorded under starvation (no food, only water added) for 12 h. After 12 h, flies were transferred on food and survival was recorded daily. (**C**) Survival was recorded for 12 h under starvation (no food) and desiccation (no water) conditions. (**D**) After 12 h of starvation and desiccation, flies were transferred onto food and survival was recorded daily. (**E**) Survival was recorded in flies injected with different doses of Sc-ShK-1 under starvation and desiccation conditions for 12 h. Log-rank test *p*-value significance indicated by asterisks using the following cut offs values: 0.0004–0.0006 *** and <0.0001 ****.

To determine the toxicity of Sc-ShK-1, 100 ng of recombinant Sc-ShK-1 protein was injected into adult *Drosophila melanogaster* and survival was counted daily. There was no significant mortality found under normal conditions where food and water are supplied *ab libitum* (Figure 2A). Next, we tested the toxicity under stressful conditions—starvation and desiccation—where flies were left in empty vials post-injection for 12 h. We observed ~50% of mortality in 12 h under starvation and desiccation conditions (Figure 2C). After 12 h, the flies were transferred back onto food and survival was monitored daily (Figure 2D). We also tested starvation alone. No significant mortality was found under starvation conditions (Figure 2B). Next, the toxicity of the protein was assessed at different doses under starvation and desiccation conditions, and a dose dependent response was observed (Figure 2E). Margatoxin (MgTx) was used as a positive control for the Sc-ShK-1 protein. MgTx is a toxin from scorpion venom that blocks both Kv1.3 and Kv1.2 channels with picomolar affinity [2,35,36]. Injecting 20 ng of MgTx into flies showed a loss of climbing phenotype that was reversible overtime (Supplementary materials). MgTx also showed significant toxicity under starvation and desiccation conditions. PBS and denatured Sc-ShK-1 were used as the null and negative controls for Sc-ShK-1 protein and did not show significant mortality under stress conditions.

### 3.3. Sc-ShK-1 Negatively Affects Host Health

**Figure 3:**
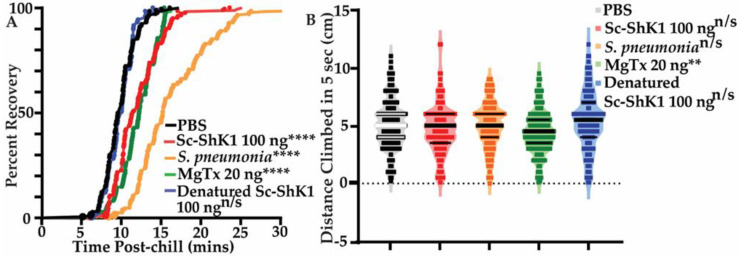
Effects of 100ng of Sc-ShK-1 on chill coma recovery and negative geotaxis. (**A**) Chill coma recovery assay. (**B**) Negative geotaxis assay was done to assess fly health. Significance indicated by asterisks (**** indicates *p*-value < 0.0001 and ** indicates *p*-value < 0.05).

In addition to survival, we used non-lethal health-sensitive behavioral assays to determine the effect of Sc-ShK-1. Previous work had shown that *Streptococcus pneumoniae* bacterial infection causes significant health effects on flies as measured by behavior [31]. We assessed chill coma recovery and negative geotaxis (climbing) to evaluate fly health after injecting 100 ng of Sc-ShK-1. We observed significant delays in recovery from the coma with Sc-ShK-1 and MgTx compared to PBS control (Figure 3A). However, negative geotaxis was not affected by Sc-ShK-1 though it did show significant decline with MgTx, likely due to its paralytic activity (Figure 3B). Here, we used *Streptococcus pneumoniae* bacterial infection as a positive control. Denatured Sc-ShK-1 was no different than the PBS control in both assays.

### 3.4. Sc-ShK-1 Modulates Host Immunity in a Bacterial Co-Infection

**Figure 4:**
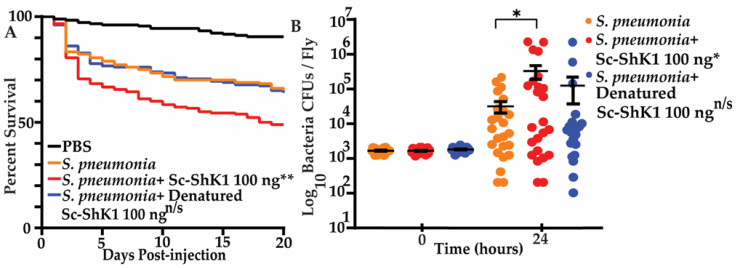
(**A**) 100 ng of Sc-ShK-1 was co-injected with the LD_20_ dose of *Streptococcus pneumonia* and survival was monitored daily. (**B**) CFUs were measure at 0 h and 24 h post-injection. Significance indicated by asterisks (** indicates *p*-value 0.0018 and * indicates *p*-value 0.0379).

To investigate the immunomodulatory function of Sc-ShK-1, we took the advantage of *Streptococcus pneumoniae* bacterial infection and measured fly survival and microbial growth [31]. Infection with *S. pneumonia* bacteria is lethal to flies in a dose-dependent manner [29]. Flies co-injected with 100 ng of Sc-ShK-1 and the LD_20_ dose (3000 colony forming units (CFUs) of *S. pneumonia* showed significantly greater mortality compared to the LD_20_ dose of *S. pneumonia* alone (Figure 4A). Bacterial loads at the time of injection and 24-h post-injection were quantified. After overnight growth, colonies were counted and CFUs were calculated. A significant increase in 24-h microbial load was observed in flies co-injected with Sc ShK-1 (Figure 4B). Denatured Sc-ShK-1 was also tested with the LD_20_ dose of *S. pneumonia.* No significant differences in survivals and CFUs were observed with denatured Sc-ShK-1.

## 4. Discussion

### 4.1. EPN Model Supports the Study of Toxin Molecules

The EPN *S. carpocapsae* releases a mixture of 472 proteins during active infection and these ESPs have been found to be lethal on their own [14]. Many of the proteins in this mixture are known toxins such as Shk-domain-containing proteins. All the functions of these ESPs have not been discovered, but they are known to be involved in tissue damage and immunosuppression of the host [14,37]. Previous studies have shown that a variety of proteins from *S. carpocapsae* ESPs are able to disrupt host immunity including proteases, serine protease inhibitors, and fatty acid- and retinol-binding proteins (FARs) [18,31,37,38,39,40,41,42]. In this study, we showed that Sc-ShK-1, one of the ShK-domain-containing proteins, has toxic and immunomodulatory effects in the host. ShK-domain-containing proteins are conserved in nematode parasites of mammals [21] (Figure 1B). The function of these proteins in infections of insects and mammals may be similar [14,43]. Therefore, EPNs are a good model system for studying the ESPs of nematodes in general. Understanding how nematode secreted ShK proteins modulate insect immunity may provide insight into the mechanism of other nematode infections.

One concern with any study of nematode ESPs or individual secreted proteins is the physiological relevance of the amount of protein used. Previous work has shown that a single *S. carpocapsae* infective juvenile (IJ) can secrete 0.49 ng of protein in 24 h in the host during an infection. Extrapolation from this data means that 20 IJs could secret 10 ng of protein [14]. Sixty nanograms of Sc-ShK-1 had significant health effects under stressful conditions (Figure 2E), which is physiologically higher than a 20-IJ EPN infection, but it is near physiological concentrations. It is also important to note that, during an active nematode infection, nematodes release hundreds of proteins in the ESPs [14]. These proteins have evolved to operate in concert rather than in isolation. It is possible the combinatorial action of these proteins is synergistic in a way that facilitates the action of Sc-ShK-1 in a host environment. Interestingly, 100 ng of Sc-ShK-1 alone had phenotypic effects at the organismal level affecting behavior as well as the outcome of a bacterial infection.

### 4.2. Sc-ShK-1 Toxin Increases Host Susceptibility during Stress Condition

It is established that the proteins and effector molecules released by parasitic nematodes play crucial role in infection [14,21,29,36]. To combat infections, hosts carry out cellular and humoral processes that help to destroy the pathogens [44]. These immune responses are complex and energetically demanding. To support the huge changes in immune signaling, hosts need to go through metabolic switches that have significant effects on physiology, development, and survival [44]. A recent study showed that the consequence of a metabolic shift due to infection has both immediate and long-term effects on the survival of the host during stressful conditions such as starvation and desiccation [44]. We utilized this principle in testing the activity of Sc-ShK-1 under stressful conditions. Our initial assessment of Sc-ScK-1 toxicity was done under normal conditions, without additional stressors such as starvation or desiccation. Under these conditions we observed that injection of Sc-ShK-1 caused no significant mortality. We also tested under starvation alone (no food, only water added) and no significant mortality was observed. When Sc-ShK-1 was injected into flies under starvation and desiccation conditions, increased mortality was observed in flies with Sc-ShK-1 protein compared to PBS control flies in 12 h. Metabolic stress revealed a toxic effect of a nematode protein that was not observed under normal conditions.

### 4.3. Chill Coma Recovery and Negative Geotaxis Assay Is Useful to Measure Fly Health

Infections cause damage and are energetically costly to fight [29,44]. To survive an infection, hosts utilize both resistance and tolerance, which depend on the fitness of the animal [29,45]. In this study we demonstrated infection related deficits using behavioral assays that allowed us to monitor fly health before death. In chill coma recovery assays, flies were exposed to cold temperatures for a short period of time. Moving back from the cold, flies gradually recover, which depends on the fitness of the flies before the cold-induced coma [29,46]. When flies are presented with an immune insult, the combined effect of insult and low temperature significantly affects fly health resulting in delayed recovery, or even death [30,46]. Negative geotaxis measures how fast flies able to climb vertically against gravity [29,47]. Sick flies tend to climb slower than healthy flies. We injected flies with either Sc-ShK-1, MgTx, or *S. pneumoniae* and left them on food for overnight to allow the immune insult to exert an effect on the fly. The following day, we performed the behavioral assays. We observed significant delays in recovery with Sc-ShK-1, MgTx, and *S. pneumoniae* infection compared to PBS controls in chill coma recovery. In negative geotaxis assays, flies injected with MgTx showed a significant decline in climbing activity compared to PBS controls. Injection with Sc-ShK-1 or *S. pneumoniae* had no significant effect on climbing. This is an interesting observation which suggests that negative geotaxis may be less sensitive in infection-related changes than the chill coma recovery assay. However, MgTx has a paralytic effect on flies which can be assessed by the negative geotaxis. 

### 4.4. Sc-ShK-1 Decreases Host Resistance to Infection

ShK-domain-containing proteins from parasitic worms may be therapeutically important because of their immunomodulatory role in effector memory T cells [2,4]. Previous work shows that two ShK-related peptides from *Ancylostoma caninum,* Acan1 and *Necator americanus,* Nak1 displayed immunomodulatory functions in human peripheral blood mononuclear cells by suppressing CD4+ T cell proliferation and inhibiting IL-2 and TNF production [4]. Though several studies were done on ShK proteins from vertebrate-parasitic nematodes, our understanding of their function and the way they modulate host immunity is incomplete. Here, we described in vivo studies of the immunomodulatory role of Sc-ShK-1 utilizing *Streptococcus pneumoniae* bacterial co-infection into fruit flies. We co-injected Sc-ShK-1 and *S. pneumoniae* bacteria into flies and monitored survival and microbial growth. Our results showed immune suppression resulting in a significant increase in microbial growth and a decrease in fly survival. In summary, Sc-ShK-1, one of the ShK-domain-containing proteins in *S. carpocapsae* ESPs actively participates in suppressing the host defense system. 

## Data Availability

All raw data are available on Mendeley. Link- Lima, Aklima; Dillman, Adler (2022), “Sc-ShK1 paper”, Mendeley Data, V1, doi: 10.17632/22pywjkx73.1.

## Supplementary Materials

The following supporting information can be downloaded at: https://www.mdpi.com/article/10.3390/pathogens11101094/s1, Figure S1: Sc-ShK-1 purification; Video S1: Mgtx causes paralysis.
