# Supplementary material for: ShK-Domain-Containing Protein from a Parasitic Nematode Modulates Drosophila melanogaster Immunity
Source: Pathogens. 2022 Sep 24;11(10):1094. doi: 10.3390/pathogens11101094 (PMC9610955; doi:10.3390/pathogens11101094)
Supplement: Supplementary file 1 [file pathogens-11-01094-s001.zip › Supplementary_Figure_1.pdf]

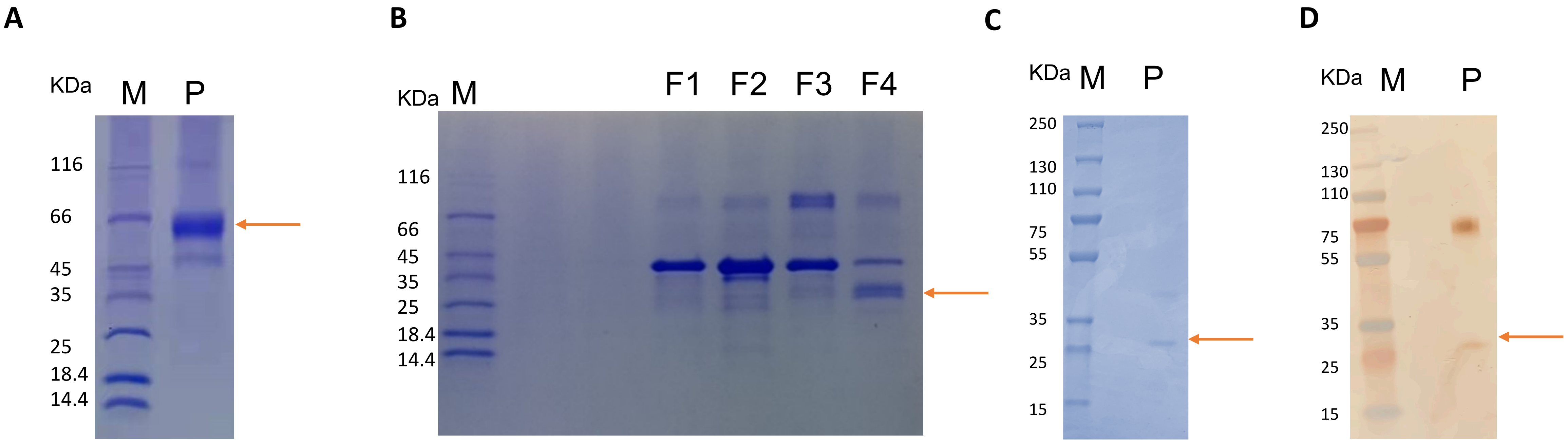

Supplementary Figure S1. Recombinant protein purification: The Sc-Shk-1 was expressed recombinantly in *Pichia pastoris* and purified **(A)** Affinity purification of fusion Sc-Shk-1 using Ni-NTA resin. **(B)** Purification of sumo protease digested fusion Sc-Shk-1 protein, F1-F4: different fractions. **(C)** Purified Sc-Shk-1 protein using size exclusion Chromatography. (D) Western blot analysis of purified Sc-Shk-1. (M: protein molecular weight ladder, P: protein). Red arrow shows the Sc-Shk-1 protein.
